# Plant-based diets and body composition in Chinese omnivorous children aged 6–9 years old: A cross-sectional study

**DOI:** 10.3389/fnut.2022.918944

**Published:** 2022-07-29

**Authors:** Gengdong Chen, Mengyang Su, Xinwei Chu, Yuanhuan Wei, Shanshan Chen, Yingyu Zhou, Zhengping Liu, Zheqing Zhang

**Affiliations:** ^1^Department of Obstetrics, Foshan Institute of Fetal Medicine, Southern Medical University Affiliated Maternal & Child Health Hospital of Foshan, Foshan, China; ^2^Guangdong Provincial Key Laboratory of Tropical Disease Research, Department of Nutrition and Food Hygiene, School of Public Health, Southern Medical University, Guangzhou, China

**Keywords:** plant-based diet, body composition, abdominal obesity, handgrip strength, children

## Abstract

Evidence suggests that plant-based diets are beneficial for alleviating metabolic diseases. Childhood is a crucial period for body growth and development. However, it is unknown whether adherence to a plant-based diet is related to a healthy body composition in children. We aimed to assess the relationship between a plant-based diet and body composition in children. A total of 452 Chinese children aged 6–9 years old participated in this cross-sectional study. Lean mass (LM), fat mass, and fat mass percentage (FMP) were assessed via dual-energy X-ray absorptiometry. An age- and sex-specific abdominal FMP ≥85th percentile was defined as abdominal obesity. Handgrip strength was measured using a hydraulic hand dynamometer. A validated 79-item food frequency questionnaire was used to collect dietary information. Overall plant-based diet index (PDI), healthful plant-based diet index (hPDI), and unhealthful plant-based diet index (uPDI) scores were calculated. After adjusting for potential covariates, a higher hPDI score (per 10-score increment) was associated with a higher LM in the android area (0.038 kg, 3.2%), gynoid area (0.048 kg, 1.9%), and trunk (0.102 kg, 1.2%) and with a lower FMP (1.18%) in the android area. In contrast, a higher uPDI score (per 10-score increment) was associated with a lower LM in the trunk (0.091 kg, 1.1%) and android area (0.023 kg, 1.9%) and with a higher FMP (0.74%) in the android area. No significant associations were observed between the overall PDI and body composition or abdominal obesity. After stratifying by sex, higher (vs. lower) hPDI scores was associated with lower abdominal obesity risk in girls and higher handgrip strength in boys. In conclusion, in this cross-sectional study, we found that stronger adherence to a healthful plant-based diet, and less adherence to an unhealthful plant-based diet was associated with better body composition in Chinese omnivorous children aged 6–9 years old. Our results highlight the need to distinguish between healthy and unhealthy plant foods within investigating how to obtain a healthy body composition in children.

## Introduction

The prevalence of obesity and/or overweight has increased sharply worldwide in recent decades to become one of the most important public health issues (1, 2), and the rate of increasing obesity and overweight prevalence is greater in children than in adults (1). Childhood adiposity can lead to obesity in adulthood and increase the risk of metabolic consequences, such as diabetes, cardiovascular events, and non-alcoholic fatty liver disease (3). Furthermore, detrimental precursor metabolic processes might occur in children with obesity or overweight, even at an early age (4). Thus, additional public health strategies to reduce the prevalence of overweight and obesity in children are urgently needed.

Nutrition is one of the most important factors that affects body composition. Previous studies have suggested that a plant-based diet, defined as a high intake of plant foods and a low intake of animal foods in an omnivorous diet, might contribute to improved body composition in adults (5–7). These findings are in agreement with those of studies that have investigated healthy dietary patterns, such as the Mediterranean diet (8) and the DASH diet (9), in adults. These diets are characterized by a high intake of healthy plant foods (e.g., fruits and vegetables). However, some plant foods are high in sugar (10) or contain refined carbohydrates (11), such as potatoes and refined grains, and therefore might be detrimental to health. To define a healthy plant-based diet and distinguish between healthy and unhealthy plant foods, Hu et al. developed three plant-based diet indexes to assess the degree of adherence to a plant-based diet: the overall plant-based diet index (PDI), the healthful plant-based diet index (hPDI), and the unhealthful plant-based diet (uPDI) (12). In adults, a higher hPDI and lower uPDI were associated with a lower risk of cardiovascular events (13), type two diabetes and gestational diabetes (12), and metabolic syndrome (14). These findings indicate the possible value of a healthy plant-based diet for preventing adiposity and adverse metabolic consequences. However, relevant studies investigating plant-based diets in children are scarce. Vegan or vegetarian diets were found to be associated with lower BMI, fat mass index, or FMP in three cross-sectional studies in Polish children, while null associations between LM and these diets were observed (15–17). In children, the diet must meet the daily physiological activity needs and maintain body growth and development, particularly in young children. Animal foods contain potentially harmful compositions (i.e., saturated fat, cholesterol) that increase the risk of chronic disease, including: coronary heart disease, and cancer (18–20). However, animal foods are also an important source of protein for the development of LM (21). A systematic review including 17 studies discover that plant based diet pattern was negatively associated with both FM and LM in middle aged and elderly population (22). In another cross-sectional study of 3,322 meat eaters and 1,186 vegetarians held in UK, compared with meat eaters, vegetarians of Indian British women had lower LM (23). No consistent results could be achieved whether adherence to a plant-based diet can improve children's body composition (lower FM and/or higher LM).

We explored the associations between plant-based diets and body composition, abdominal obesity, and handgrip strength in a cross-sectional study of Chinese omnivorous children aged 6–9 years old.

## Materials and methods

### Participants

This cross-sectional study was conducted in children 6–9 years of age in Guangzhou City, Guangdong Province, China. It was carried out from December 2015 to March 2017; the recruitment procedure has been previously described (24). Briefly, 521 of 1,600 children agreed to participate in the study. The participants were recruited through invitation letters, advertisements, and personal referrals. Among them, 69 children were excluded for the following reasons: preterm birth, a twin, a relevant medical condition (e.g., digestive tract disease, kidney stones or nephritis, thyrotoxicosis, hepatitis, anaphylactoid purpura, and metabolic bone disease), or core data were missing. A final sample of 452 singleton children (255 boys and 197 girls) was included in the analysis. The children voluntarily participated in the study and received a free general physical examination and a dual energy X-ray absorptiometry (DXA) scan, with permission from and accompanied by their parent or legal guardian. Before the examination, the study procedures and consent were explained to the guardian and child, and written consent was obtained from each guardian on behalf of the child participant. This cross-sectional study was carried out in accordance with the Declaration of Helsinki and was approved by the ethics committee of the School of Public Health at Sun Yat-sen University (no. 201549).

### Dietary information

Through face-to-face interviews, dietary data for the prior year were obtained from each participant using a 79-item food frequency questionnaire (FFQ), which was the same as used in the Guangzhou Nutrition and Health Study (25). The FFQ used had been previously validated in our population (26). The FFQ consisted of 79 food items, grouped under subheadings like cereals (12 food items), soy and beans (8 items), vegetables (total: 13 items; leafy vegetable: 6 items; melon and fruit vegetable: 4 items; root vegetable: 3 items), fruits (10 items), meats (total: 17 items; livestock meat or visceral organ: 7 items; poultry meat: 3 items; fish: 4 items; other seafoods: 3 items), eggs (1 items), dairy products (8 items), Fungous and nuts (2 items), and beverage and drinks (8 items). The food items were organized on the basis of cultural use and physical composition. Colorful photographs of standard food portion sizes were used to help estimate the exact quantity of each food. The children reported their consumption frequencies (never; yearly; monthly; weekly; or daily) and the estimated average amount of each food item per time over the last 12 months with the help of their guardian/caregiver, then FFQs were filled out by the children in conjunction with their guardians/caregiver. We used the Chinese Food Composition (2009) (27) to calculate the mean daily intake of energy and other nutrients. To attenuate the influence of energy, the daily intakes of specific food groups were adjusted for energy using the residual method.

Hu et al. developed three plant-based diet indexes to assess the degree of adherence to a plant-based diet: the overall plant-based diet index (PDI), the healthful plant-based diet index (hPDI), and the unhealthful plant-based diet index (uPDI) (12). Eighteen food groups classified into three broad categories (healthy plant foods, unhealthy plant foods, and animal foods) are used to calculate the PDIs. Fruits, whole grains, vegetables, vegetable oils, legumes, nuts, and tea/coffee are defined as healthy plant foods. Refined grains, potatoes, fruit juices, sugar-sweetened beverages, and sweets/desserts are defined as unhealthy plant foods. Animal fats (including butter added to food and butter or lard used for cooking), dairy products, seafood/fish, eggs, meat (including red/processed meat and poultry), and miscellaneous animal-based foods are included in the animal food group. More detail information of the original description of these food groups could be found in the Appendix table of the article of Satija et al. (12). In our study, sweets/desserts, animal fats, and miscellaneous animal-based foods were not included in the FFQ and were therefore excluded; the remaining 15 food groups were included in the PDI score calculations. These 15 food groups were ranked in sex-specific quintiles and assigned a score ranging from 1 to 5. For the overall PDI, higher scores were given to the participants with higher intakes of plant food groups and lower intakes of animal food groups. For the hPDI, higher scores were given to those with higher intakes of healthy plant food groups, lower intakes of unhealthy plant food groups and animal food groups. For the uPDI, unhealthy plant food groups received positive scores, whereas healthy plant and animal food groups received reverse scores. The quintile scores of each food group were summed to obtain the final PDI scores (overall PDI, hPDI, or uPDI), which ranged from 15 to 75 in this study. A higher PDI score indicated stronger adherence to a specific plant-based diet. Examples of foods constituting the healthy plant food groups, unhealthy plant food groups, and animal food groups in this study were presented in Supplementary Table 1.

### DXA scans and handgrip strength

Body mass index (BMI) is a simple measure that is widely used to evaluate a person's adiposity status. However, body composition analysis can better distinguish fat and lean mass and can be used to examine their distributions within the body, which provides a better evaluation of adiposity. A higher lean mass and a lower fat mass and fat mass percentage (FMP) indicates a healthier body composition and is associated with a lower risk of metabolic diseases (28).

A whole-body DXA scanner (Discovery W; Hologic Inc., Waltham, MA, U.S.A.) was used to assess the participants' body composition. Experienced technicians operated the scanner and processed the resulting data. For the scan, the participants were asked to wear light clothing and to not wear metal or other high-density objects. The participants held a standard lying posture during the scan with the help of a technician. The lean mass (LM) and fat mass (FM) at multiple sites (whole body, trunk, limbs, android area, and gynoid area) were analyzed, and the FMP was calculated as follows: FMP = 100% × FM/LM. To assess measurement variation, we conducted two consecutive measurements with repositioning in 35 randomly selected children, and the coefficients of variation (CV) were 0.77–5.67% for the LM and FM across the multiple sites. Fat can accumulate in different subcutaneous area of the body, and two different obesity phenotypes were classified. Fat deposition in the android area known as central or abdominal obesity, while fat deposition in the gynoid area known as peripheral obesity. Compared with peripheral (gynoid) obesity, abdominal (android) obesity confers increases risk of metabolic complications (29). Evidence also showed fat distribution in the android area, rather than gynoid region may be important factor in determining the risk of cardiovascular disease in the National Health and Nutrition Examination Survey 2003–2006 of America (30). Therefore, we aimed to explore the associations of PDIs and abdominal obesity in this study. An age- and sex-specific abdominal FMP (in the android area) ≥85th percentile was defined as abdominal obesity as previously described (24).

A Jamar^®^ Plus+ hand dynamometer (Sammons Preston, Bolingbrook, IL, U.S.A.) was used to measure the children's handgrip strength, and the data were accurate to 0.1 kg. The children performed the measurement twice with a short break in between using both hands in a standing posture; the highest handgrip strength was used for the analyses. Twenty-eight randomly selected children repeated the handgrip strength test after a 30 min interval. The CVs were 8.2% for the right hand and 9.5% for the left hand.

### Potential covariates

The height and weight were measured with the children wearing light clothing and without shoes. The data were accurate to 0.1 cm for height and to 0.1 kg for weight. Face-to-face interviews using a structured questionnaire were performed to collect information about potential covariates over the last year of investigation, including age (years), birth information (vaginal delivery or cesarean section), household income (≤150,00 or >150,00 Yuan/month), maternal (≤12 or >12 years) and paternal education (≤12 or >12 years), and the supplemental use of calcium (yea or not) or multivitamin tablets (yea or not). The participants' physical activity was assessed using a continuous 3-day (2 weekdays and 1 weekend day) record for the prior week, which investigated the daily physical activities that children were engaged in, and time expenditure of each items (accurate to 15 min) as previously described (24).

### Statistical analysis

The PDI scores were calculated and divided into sex-specific tertiles, with higher tertiles representing stronger adherence to one of the three PDIs. The characteristics of the participants in the bottom and top PDI tertiles are presented. Continuous variables are presented as the mean ± standard deviation or median (interquartile range). Categorical variables are presented as frequencies (percentages). Linear regression analysis was performed to explore the associations between the PDIs (per 10-score increment) and the body composition at multiple sites and handgrip strength. Logistic regression analysis was used to identify associations between the PDIs (per 10 scores increment or comparison between tertiles) and abdominal obesity (abdominal FMP ≥85th percentile). Two adjustment models were carried out; Model 1 was a univariate model, and Model 2 was a multivariate model adjusted for age, sex, delivery method, height, weight, household income, maternal and paternal education, supplemental use of calcium or multivitamin tablets, physical activity, and dietary intake of energy. According to former and the researcher's experiences, the covariates were introduced into the analysis using the “Enter” model in order to control possible confounding. Sensitivity analysis stratified by sex was also performed. In order to compare body composition between top tertile groups of hPDI and uPDI, analyses of covariates (ACNOVA analysis) were carried out with the adjustment of covariates in Model 2. The statistical analyses were conducted using SPSS 21.0 (SPSS Inc., Chicago, IL, U.S.A.). Statistical significance was defined as a two-sided *P*-value of <0.05.

## Results

A total of 452 children with a mean age of 8.0 ± 0.9 years were included in this study. The participants with higher overall PDI, hPDI, or uPDI scores had stronger adherence to an overall plant-based diet, healthful plant-based diet, or unhealthful plant-based diet, respectively. Detail characteristic information and dietary information of daily intake of different foods were displayed in Tables 1, 2, respectively. The children with a higher overall PDI score tended to weigh more and to have higher maternal education, higher consumption of healthy and unhealthy plant foods, lower household income, lower paternal education, less use of calcium supplements, and lower consumption of animal foods. The children with a higher hPDI score tended to weigh less and to have higher maternal and paternal education, more use of calcium supplements, higher intake of healthy plant foods, and lower household income. The children with a higher uPDI score tended to be born through cesarean section and to have lower household income, lower parental education, less use of calcium and multivitamin supplements, lower consumption of healthy plant foods and animal foods, and higher intake of unhealthy plant foods.

**Table 1:** Characteristic of subjects included in the study.

		**PDI**		**hPDI**		**uPDI**	
		***T1***	***T3***	***T1***	***T3***	***T1***	***T3***
Subjects, *N*		147	149	159	150	146	157
Mean scores		38.9 ± 3.23	51.6 ± 3.26	39.8 ± 2.62	51.0 ± 2.41	38.2 ± 2.57	51.1 ± 3.18
Age, years		7.94 ± 0.96	8.14 ± 0.85	8.04 ± 0.94	8.02 ± 0.95	7.93 ± 0.95	8.02 ± 0.98
Height, cm		128 ± 8.26	130 ± 7.63	129 ± 7.61	129 ± 8.39	128 ± 8.39	129 ± 8.27
Weight, kg		26.1 ± 6.60	26.9 ± 6.88	26.4 ± 6.62	25.7 ± 6.65	26.6 ± 7.52	26.7 ± 7.18
Physical activity, Met × h/d		39.8 ± 4.38	40.0 ± 4.14	40.0 ± 4.40	40.2 ± 4.43	40.2 ± 3.89	39.6 ± 4.30
Sex	Girls	63 (42.9)	68 (45.6)	68 (42.8)	66 (44.0)	63 (43.2)	70 (44.6)
	Boys	84 (57.1)	81 (54.4)	91 (57.2)	84 (56.0)	83 (56.8)	87 (55.4)
Delivery way, *N (%)*	Natural	74 (50.3)	77 (51.7)	79 (49.7)	73 (48.7)	71 (48.6)	85 (54.1)
	Cesarean	73 (49.7)	72 (48.3)	80 (50.3)	77 (51.3)	75 (51.4)	72 (45.9)
Household income, Yuan × month^−1^, *N* (%)	≤150,00	66 (44.9)	75 (50.3)	73 (45.9)	75 (50.0)	68 (46.6)	79 (50.3)
	>150,00	81 (55.1)	74 (49.7)	86 (54.1)	75 (50.0)	78 (53.4)	78 (49.7)
Maternal education, *N* (%)	≤12 years	58 (39.5)	56 (37.6)	62 (39.0)	54 (36.0)	50 (34.2)	69 (43.9)
	>12 years	89 (60.5)	93 (62.4)	97 (61.0)	96 (64.0)	96 (65.8)	88 (56.1)
Paternal education	≤ 12 years	55 (37.4)	66 (44.3)	64 (40.3)	55 (36.7)	45 (30.8)	74 (47.1)
	>12 years	92 (62.6)	83 (55.7)	95 (59.7)	95 (63.3)	101 (69.2)	83 (52.9)
Use of calcium supplements, *N* (%)	No	82 (55.8)	95 (63.8)	99 (62.3)	86 (57.3)	83 (56.8)	100 (63.7)
	Yes	65 (44.2)	54 (36.2)	60 (37.7)	64 (42.7)	63 (43.2)	57 (36.3)
Use of multi-vitamin supplements, *N* (%)	No	123 (83.7)	124 (83.2)	130 (81.8)	121 (80.7)	118 (80.8)	135 (86.0)
	Yes	24 (16.3)	25 (16.8)	29 (18.2)	29 (19.3)	28 (19.2)	22 (14.0)

*Continuous variables were presented as Mean ± standard deviation; Categorical variables were presented as frequency (percentage). a, adjusted for energy using residual methods*.

**Table 2:** Dietary information of intake of different foods in our study.

	**PDI**		**hPDI**		**uPDI**	
	***T1***	***T3***	***T1***	***T3***	***T1***	***T3***
Subjects, *N*	147	149	159	150	146	157
Total energy intake, kcal/d	1,450 ± 419	1,440 ± 425	1,441 ± 423	1,390 ± 443	1,411 ± 430	1,441 ± 454
**Healthy plant foods**						
Whole grains, g/d	0.0 (0.0, 2.0)	1.5 (0.0, 5.2)	0.0 (0.0, 1.6)	1.9 (0.0, 5.5)	1.7 (0.0, 5.3)	0.0 (0.0, 1.9)
Fruits, g/d	102 (68, 170)	142 (99, 220)	88 (58, 138)	162 (113, 219)	151 (111, 216)	87 (58, 143)
Vegetables, g/d	140 (99, 209)	186 (134, 241)	149 (111, 190)	202 (133, 254)	209 (159, 266)	143 (108, 188)
Nuts, g/d	2.9 (0.8, 7.7)	5.4 (2.6, 12)	2.6 (0.9, 5.3)	7.9 (3.3, 15)	6.3 (2.6, 15)	2.5 (0.9, 5.8)
Legumes, g/d	4.2 (2.0, 7.3)	9.4 (5.3, 16)	4.6 (2.1, 7.5)	9.7 (5.7, 17)	7.4 (3.7, 13.3)	4.9 (2.2, 9.2)
Vegetable oils, g/d	16 (10, 26)	21 (15, 33)	17 (10, 28)	20 (15, 32)	20 (11, 33)	20 (10, 25)
Tea and coffee, g/d	0.0 (0.0, 0.0)	0.0 (0.0, 0.0)	0.0 (0.0, 0.0)	0.0 (0.0, 0.0)	0.0 (0.0, 0.0)	0.0 (0.0, 0.0)
**Unhealthy plant foods**						
Fruits juices, g/d	2.4 (0.0, 11)	16 (7.6, 29)	14 (5.4, 31)	4.1 (0.0, 15)	5.0 (0.0, 15)	15 (4.2, 36)
Refined grains, g/d	146 (118, 166)	164 (133, 186)	161 (140, 185)	147 (125, 174)	130 (112, 147)	179 (158, 201)
Potatoes, g/d	8.5 (4.2, 13.8)	18 (12, 30)	14 (7.6, 26)	12 (6.8, 22)	14 (7.8, 23)	15 (7.6, 26)
Sugar-sweetened beverages, g/d	3.0 (0.0, 12)	14 (3.5, 26)	14 (5.3, 33)	1.3 (0.0, 7.5)	1.2 (0.0, 6.4)	14 (3.2, 31)
**Animal foods**						
Dairy, g/d	259 (168, 367)	151 (104, 227)	228 (159, 318)	169 (113, 271)	239 (157, 357)	174 (114, 275)
Eggs, g/d	34 (21, 47)	23 (12, 35)	32 (19, 45)	26 (14, 40)	36 (25, 50)	23 (15, 32)
Fish or seafood, g/d	25 (13, 46)	20 (8.8, 32)	28 (13, 44)	17 (9.4, 31)	28 (15, 48)	15 (6.8, 26)
Meat, g/d	124 (93, 156)	86 (60, 110)	113 (80, 150)	96 (70, 125)	120 (87, 153)	90 (60, 125)

As shown in Table 3, after adjusting for potential covariates, no significant associations were found between the overall PDI score and body composition (FM, LM, or FMP). A higher (vs. lower) hPDI score was associated with higher LM and lower FMP, whereas a higher (vs. lower) uPDI score was associated with lower LM and higher FMP, especially for the android area composition. Each 10-score increment in the hPDI score was associated with increment LMs in the areas of android area (0.038-kg, 3.2% of the mean), trunk area (0.102-kg, 1.2% of the mean), and the gynoid are (0.048-kg, 1.9% of the mean). Besides, each 10-score increment in the hPDI score was associated with a 1.18% (4.9% of the mean) decrease in the android area FMP. In contrast, each 10-score increment in the uPDI score was associated with a 0.023-kg (1.9% of the mean) decrease in the android area LM, a 0.091-kg (1.1% of the mean) decrease in the trunk area LM, and a 0.74% (3.0% of the mean) increment in the android area FMP. After stratification by sex, for girls, a higher hPDI score was associated with an increased android and gynoid area LM and a decreased android area FMP. In girls, an increased uPDI score was associated with a decreased gynoid area LM (Supplementary Table 2). In boys, a higher hPDI score was associated with an increased android and trunk area LM and with a decreased android and gynoid area FMP. A higher uPDI score in boys was associated with a decreased android and trunk area LM and an increased android area FMP (Supplementary Table 2).

**Table 3:** Associations of plant-based diet index scores with body composition after adjusted for potential covariates.

**Body composition**	**Per 10-score increment of plant-based diet index**								
	**PDI**			**hPDI**			**uPDI**		
	***β***	***se***	***p***	***β***	***se***	***p***	***β***	***se***	***p***
**Whole body**									
FM, kg	−0.100	0.086	0.244	−0.155	0.097	0.113	0.052	0.087	0.551
LM, kg	0.098	0.084	0.246	0.155	0.095	0.103	−0.125	0.085	0.140
FMP, %	−0.408	0.309	0.188	−0.683	0.349	0.051	0.378	0.311	0.226
**Trunk**									
FM, kg	−0.009	0.043	0.830	−0.046	0.048	0.343	0.024	0.043	0.570
LM, kg	0.036	0.043	0.399	0.102	0.048	0.035	−0.091	0.043	0.035
FMP, %	−0.251	0.328	0.444	−0.672	0.370	0.070	0.495	0.330	0.134
**Limbs**									
FM, kg	−0.091	0.052	0.081	−0.102	0.059	0.085	0.024	0.053	0.654
LM, kg	0.064	0.049	0.185	0.076	0.055	0.171	−0.040	0.049	0.415
FMP, %	−0.670	0.424	0.115	−0.886	0.480	0.066	0.375	0.429	0.382
**Android area**									
FM, kg	−0.003	0.008	0.676	−0.007	0.009	0.446	0.005	0.008	0.545
LM, kg	0.007	0.010	0.436	0.038	0.011	<0.001	−0.023	0.010	0.016
FMP, %	−0.440	0.345	0.203	−1.183	0.388	0.002	0.742	0.347	0.033
**Gynoid area**									
FM, kg	−0.018	0.017	0.289	−0.018	0.019	0.364	0.016	0.017	0.343
LM, kg	0.019	0.019	0.317	0.048	0.021	0.024	−0.034	0.019	0.075
FMP, %	−0.590	0.382	0.123	−0.827	0.432	0.056	0.628	0.385	0.103

*FM, fat mass; LM, lean mass; FMP, fat mass percentage*.

*Linear regression analysis, adjusted for covariates including: age, sex, height, weight, delivery way, household income, parental education, physical activity, use of calcium and multi-vitamin supplements, dietary intake of energy. The bold values indicates the statistical significance (p < 0.05)*.

We further compared body composition between top tertile groups of hPDI and uPDI scores. As shown in Table 4, compared with subjects in top uPDI tertiles, those with top hPDI tertiles scores tended to be with lower FMP at sites of whole body (0.99%), trunk (1.15%), android area (1.38%), and gynoid area (1.38%); and be with higher LM at sites of trunk (0.23 kg) and android area (0.05 kg).

**Table 4:** Comparison of body composition between top tertile groups of hPDI and uPDI scores.

**Body composition**	**Top tertile of hPDI (*****N*** = **134)**		**Top tertile of uPDI (*****N*** = **142)**		**Difference** ^a^	***P*** **-value of difference**
	***Mean***	***Se***	***Mean***	***Se***		
**Whole body**						
FM, kg	7.427	0.105	7.555	0.102	−0.128	0.388
LM, kg	19.036	0.153	18.652	0.149	0.384	0.076
FMP, %	26.901	0.301	27.890	0.292	−0.989	0.021
**Trunk**						
FM, kg	2.795	0.054	2.855	0.052	−0.060	0.434
LM, kg	8.432	0.076	8.204	0.074	0.228	0.034
FMP, %	23.503	0.330	24.651	0.320	−1.148	0.014
**Limbs**						
FM, kg	3.809	0.059	3.878	0.057	−0.070	0.183
LM, kg	7.844	0.077	7.698	0.075	0.146	0.401
FMP, %	31.222	0.419	32.356	0.407	−1.134	0.056
**Android area**						
FM, kg	0.413	0.009	0.419	0.009	−0.005	0.696
LM, kg	1.218	0.014	1.167	0.013	0.050	0.010
FMP, %	23.509	0.342	24.886	0.332	−1.377	0.005
**Gynoid area**						
FM, kg	1.275	0.020	1.300	0.019	−0.025	0.369
LM, kg	2.563	0.029	2.486	0.029	0.077	0.065
FMP, %	32.155	0.381	33.537	0.370	−1.381	0.011

*FM, fat mass; LM, lean mass; FMP, fat mass percentage*.

*ANCOVA analyses, adjusted for covariates including: age, sex, height, weight, delivery way, household income, parental education, physical activity, use of calcium and multi-vitamin supplements, dietary intake of energy*.

a *Difference = Mean _(Top tertile of hPDI)_ – Mean _(Top tertile ofuPDI)_. The bold values indicates the statistical significance (p < 0.05)*.

We further explored the relationships between the plant-based diet indexes and abdominal obesity in children (Table 5). Although the children with a higher hPDI score (higher tertile or per 10-score increment in the hPDI score) tended to exhibit less abdominal obesity, and those with higher PDI and uPDI scores tended to exhibit more abdominal obesity, these associations did not reach statistical significance (*P*-values of 0.105 to 0.637). After stratifying by sex (Supplementary Table 3), in girls, there was a significant negative association between the highest hPDI scores and lower abdominal obesity (OR = 0.02, 95% CI: 0001, 0.48).

**Table 5:** Associations of plant-based diet index scores with abdominal obesity.

**Abdominal obesity**	**Plant-based diet index**									
	***T1***	***T2***			***T3***			***Per 10 increment***		
	***Reference***	***OR***	***95%CI***	***p***	***OR***	***95%CI***	***p***	***OR***	***95%CI***	***p***
**PDI**										
Model 1	1.00	1.16	(0.65, 2.09)	0.616	1.23	(0.68, 2.21)	0.490	1.12	(0.75, 1.68)	0.589
Model 2	1.00	0.84	(0.34, 2.05)	0.693	1.24	(0.51, 2.97)	0.637	1.27	(0.68, 2.37)	0.457
**hPDI**										
Model 1	1.00	1.34	(0.77, 2.33)	0.298	0.70	(0.38, 1.29)	0.251	0.76	(0.48, 1.21)	0.244
Model 2	1.00	0.97	(0.42, 2.22)	0.941	0.75	(0.30, 1.91)	0.548	0.70	(0.34, 1.43)	0.326
**uPDI**										
Model 1	1.00	0.93	(0.50, 1.72)	0.815	1.44	(0.82, 2.54)	0.209	1.17	(0.78, 1.75)	0.441
Model 2	1.00	1.80	(0.68, 4.77)	0.238	2.12	(0.86, 5.26)	0.105	1.52	(0.82, 2.84)	0.185

*Logistic regression analysis, with Model 1 as univariate analysis without adjustment; and Model 2 adjusted for covariates including: age, sex, height, weight, delivery way, household income, parental education, physical activity, use of calcium and multi-vitamin supplements, dietary intake of energy*.

Associations between the plant-based diet indexes and handgrip strength were also investigated. For the total participant sample and for girls, there were no significant associations between handgrip strength and any of the three plant-based diet indexes (PDI, hPDI, and uPDI). After adjusting for potential covariates, a higher (vs. lower) hPDI score was positively associated with increased handgrip strength in boys (Table 6); every 10-score increment in the hPDI score was associated with a 0.61-kg (6.0% of the mean) increment in handgrip strength (mean: 10.1 kg).

**Table 6:** Associations of plant-based diet index scores with handgrip strength.

**Handgrip strength, kg**	**Per 10-score increment of plant-based diet index**								
	**PDI**			**hPDI**			**uPDI**		
	β	***se***	***p***	β	***se***	***p***	β	***se***	***p***
**Total (*N* = 452)**									
Model 1	0.34	0.23	0.143	0.16	0.26	0.540	−0.13	0.23	0.584
Model 2	0.08	0.16	0.627	0.18	0.18	0.310	−0.11	0.16	0.482
**Girls (*N* = 197)**									
Model 1	0.01	0.30	0.751	−0.06	0.31	0.843	−0.06	0.31	0.843
Model 2	−0.14	0.22	0.522	−0.14	0.23	0.538	−0.15	0.23	0.507
**Boys (*N* = 255)**									
Model 1	0.53	0.33	0.107	0.45	0.41	0.274	0.45	0.41	0.274
Model 2	0.25	0.22	0.260	0.61	0.27	0.024	−0.12	0.22	0.595

*Logistic regression analysis, with Model 1 as univariate analysis without adjustment; Model 2 adjusted for covariates including: age, sex, height, weight, delivery way, household income, parental education, physical activity, use of calcium and multi-vitamin supplements, dietary intake of energy. The bold values indicates the statistical significance (p < 0.05)*.

## Discussion

In this cross-sectional study, based on data of Chinese omnivorous children aged 6–9 years old, stronger adherence to a healthy plant-based diet (i.e., a higher hPDI score vs. lower hPDI score) was associated with a higher LM and a lower FMP in several body areas, particularly the android area. In contrast, greater consumption of an unhealthy plant-based diet (i.e., a higher uPDI score vs. lower uPDI score) was associated with less LM in the trunk and android areas and a higher FMP in the android area. After stratification by sex, higher (vs. lower) hPDI scores were associated with lower abdominal obesity risk in girls and higher handgrip strength in boys.

Stronger adherence to a healthy plant-based diet associated with better body composition in Chinese omnivorous children aged 6–9 years old in our study. Few related studies have been conducted in children. In a cross-sectional study of Polish children aged 5–10 years old, a vegan (but not vegetarian) diet was associated with a lower BMI and fat mass index compared with an omnivore diet (17). In two other cross-sectional studies performed in Poland, a vegetarian diet was associated with a lower FMP (19.1% vs. 21.2%, *p* = 0.050) and fat mass index (2.67 kg/m^2^ vs. 2.99 kg/m^2^, *p* = 0.044) in prepubertal children (15) and with a lower fat mass-to-lean mass ratio in children (16). More studies have been carried out in adults; however, firm conclusions cannot be made. In the Rotterdam Study (prospective, 7.1 years follow-up), which included 9,633 middle-aged and older adult participants, a higher PDI was associated with a lower BMI, FM, and FMP (6). A higher hPDI score (per 10-score increment) was associated with a 0.12% decrease in FMP in a cross-sectional study of 260 healthy U.S. women (5). A systematic review showed that a plant-based diet tended to be negatively associated with both FM and LM, but the results were inconclusive (22). In contrast to our study, previous studies have tended to find negative (22, 23, 31) or null associations (7, 32–35) between LM and a plant-based diet in adults and children (15–17). Possible reasons for these conflicting results are that the previous studies (1) had relatively small sample sizes, (2) did not adjust for covariates, (3) measured whole body composition rather than the composition at specific sites in the body, and (4) different measurements of diet. Additionally, the participants in our study were not strictly vegan; thus, we were unable to observe any potential negative association between a vegan diet and FM and any potential negative associations between a vegan diet and LM. We also distinguished between healthy and unhealthy food groups in our study, which was not done in the above-mentioned studies. This partitioning might have increased our ability to identify different direction of associations for the hPDI and uPDI. The need to distinguish between healthy and unhealthy plant foods has been indicated in previous studies in relation to multiple diseases (13, 36, 37). Taken together, the evidence indicates that a healthy plant-based diet, rather than an unhealthy plant-based diet, might be associated with better LM. Nevertheless, additional high-quality studies are needed to further explore this issue.

In our study, associations with higher significance for the android area were identified, and these results are supported by several previous studies. In a 14-week low-fat plant-based diet intervention in overweight postmenopausal women, the diet was associated with a smaller waist circumference but not associated with LM or FMP (34). In another study, a low-fat vegan diet intervention led to decreases in visceral fat volume (38). Compared with vegetarians, omnivores were found to have 50% greater tumor necrosis factor mRNA expression in abdominal fat, which increases adipose tissue inflammation (39). In our study, a negative association was found between a healthy plant-based diet and abdominal obesity in girls. Together, these findings emphasize the potential associations of a plant-based diet for lowering risk of abdominal obesity and the necessity for future studies to explore the detailed distribution of body composition.

Stronger hPDI adherence was associated with greater handgrip strength in boys. This result is supported by a systematic review that found that a plant-based diet was positively associated with muscle strength in middle-aged and older adults (22). Compared with an omnivore diet, a vegan diet was associated with better activity performance (higher estimated maximal oxygen consumption and submaximal endurance time to exhaustion) in physically active lean women in Canada (32). Conversely, in British Indians, those adhering to a vegetarian diet had lower handgrip strength than those adhering to an omnivore diet (23). Thus, firm conclusions cannot be made, and the effects of a healthy plant-based diet on handgrip strength require further investigation.

Subjects with higher hPDI scores tended to be with better maternal and paternal education, and follow a healthier lifestyle, like more physical activity, more use of calcium supplements. These factors might partly help to explain the positive associations of hPDI and LM and negative associations of hPDI and FMP. Although we tried to control these covariates in our analyses, the possible influence of these factors might could not be totally eliminated and still existed. Besides, several biological mechanisms might be involved for further explanations of the associations between hPDI and body composition. In children, a vegetarian diet was related to a higher ratio of anti-inflammatory to pro-inflammatory adipokines (15). Stronger hPDI adherence was associated with lower leptin levels, whereas weaker adherence was associated with high levels of high-sensitivity C reactive protein (40). A low-fat vegan diet intervention led to lower intramyocellular and hepatocellular lipid levels and improved insulin resistance (38). Transitioning to a vegan diet supplemented with fish lowered the plasma levels of branched-chain amino acids, which contribute to obesity and insulin resistance (41). Finally, a plant-based diet might be related to improved gut microbiota symbiosis and increased beneficial metabolites (e.g., short-chain fatty acids and trimethylamine N-oxide) (42).

Few studies have investigated the associations between a plant-based diet and body composition in children. A strength of our study was that we distinguished between different plant-based diet patterns (an overall, healthy, and unhealthy plant-based diet). The findings reveal that choosing healthy plant foods and avoiding unhealthy plant foods were associated with better body composition in Chinese omnivore children aged 6–9 years old. The use of gold-standard body composition measurement at multiple sites, along with measuring abdominal obesity and handgrip strength, provided comprehensive outcome information, enabling us to better understand the potential relationships between body composition and a plant-based diet. Finally, we controlled for several potential covariates in the analyses to avoid potential confounding. However, our study had several limitations. First, owing to the cross-sectional study design, we were able to identify associations but could not attribute causality. Second, few of the children in the healthy plant food group consumed tea/coffee, and sweets/desserts, animal fats, and miscellaneous animal-based foods in the unhealthy plant food group were excluded because they were not covered by our FFQ. These factors might have attenuated our ability to identify significant associations for the hPDI and uPDI. Although the associations might be underestimated, we did find statistical associations between the hPDI or uPDI score and body composition. Third, the abdominal obesity defined by an abdominal FMP ≥85th percentiles of the population in our study is sample based. Not standard reference data of abdominal obesity based on abdominal FMP data from representative sample could be found in Chinese children yet. Further studies with more representative and large sample size using standard reference of abdominal FMP data were encouraged for further examination of our results. Finally, considering our results were based on a specific population and with a small sample size, it is unclear yet if these results would translate to other age groups during adolescents or other races, ethnicities, or countries. Therefore, our finding should be interpreted with caution. Further prospective studies with larger sample sizes should be conducted to verify our results.

## Conclusion

In this cross-sectional study, stronger adherence to a healthy plant-based diet tended to be associated with a higher LM and a lower FMP in Chinese omnivorous children aged 6–9 years old, and it associated with lower risk of abdominal obesity in girls and higher handgrip strength in boys. In contrast, an unhealthy plant-based diet (higher uPDI score vs. lower uPDI score) was associated with a lower LM and a higher FMP, especially in the android area. These results highlight the need to distinguish between healthy and unhealthy plant foods to maintain a healthy body composition in young children. More prospective studies with larger sample sizes based on different populations were encouraged to be conducted to verify our results in the future.

## Data availability statement

The raw data supporting the conclusions of this article will be made available by the authors, without undue reservation.

## Ethics statement

The studies involving human participants were reviewed and approved by Ethics Committee of the School of Public Health at Sun Yat-sen University (no. 201549). Written informed consent to participate in this study was provided by the participants' legal guardian/next of kin.

## Author contributions

GC and MS analyzed the data and wrote the paper. XC, YW, SC, and YZ were parts of the data collection team. ZL and ZZ revised the manuscript. ZZ designed the project, supervised the study, and revised the manuscript. All authors contributed to the article and approved the submitted version.

## Funding

This work was supported by National Natural Science Foundation of China (No. 81502798, ZZ), Natural Science Foundation of Guangdong Province, China (No. 2015A030310399, ZZ), the Maternal and Children Nutrition and Care Fund of Biostime (No. BINCMYF15006, ZZ), and National Natural Science Foundation of China (No. 82103855, GC), Basic and Applied Basic Research Foundation of Guangdong Province (No. 2019A1515110163, GC), and the Foundation of Bureau of Science and Technology of Foshan City (No. 2220001004104, GC). The funding sponsors had no role in the design of the study; in the collection, analyses, or interpretation of data; in the writing of the manuscript, and in the decision to publish the results.

## Conflict of interest

The authors declare that the research was conducted in the absence of any commercial or financial relationships that could be construed as a potential conflict of interest.

## Publisher's note

All claims expressed in this article are solely those of the authors and do not necessarily represent those of their affiliated organizations, or those of the publisher, the editors and the reviewers. Any product that may be evaluated in this article, or claim that may be made by its manufacturer, is not guaranteed or endorsed by the publisher.
