# Supplementary material for: Plant-based diets and body composition in Chinese omnivorous children aged 6–9 years old: A cross-sectional study
Source: Front Nutr. 2022 Jul 29;9:918944. doi: 10.3389/fnut.2022.918944 (PMC9372333; doi:10.3389/fnut.2022.918944)
Supplement: Supplementary file 1 [file Table_1.DOCX]

Supplemental Table 1: Examples of foods constituting the healthy plant food groups, unhealthy plant food groups, and animal food groups in the FFQ used in this study.

| ***Plant Food Groups*** | |
| --- | --- |
| ***Healthy*** |  |
| **Whole grains** | Whole-wheat bread; oats; oat meal; corn flakes; corns; |
| **Fruits** | Orange; grapefruit; citrus; apple; pear; peach; pineapple; plum; banana; grape; litchi; longan; mango; persimmon; pawpaw; hami melon; watermelon; muskmelon; durian; guava; dragon fruit; mangosteen, other fruits; |
| **Vegetables** | Chinese flowering cabbage; Chinese broccoli; broccoli; pakchoi; leaf lettuce; lettuce; spinach; amaranth; water spinach; fragrant-flowered garlic;  Chinese cabbage; baby Chinese cabbage; cauliflower; celery; onion; green Chinese onion; garlic; tomato; white gourd; bitter gourd; towel gourd; eggplant; pumpkin; cucumber; white radish; carrot; pepper; |
| **Nuts** | Peanut; cashew nut; walnut; almond; pistachio nuts; sesame; |
| **Legumes** | Firm tofu; soft tofu; skin of tofu; soybean milk; jellied bean curd; soybean; mung bean; ormosia bean; kidney bean;pea; broad been; sweet broad pea; green bean; other beans; |
| **Vegetable oils** | Vegetable oil used for cooking; |
| **Tea & Coffee** | Coffee; tea; |
| ***Less healthy or unhealthy*** | |
| **Fruit juices** | Orange juice; apple juice; grape juice; coconut milk; other juice; |
| **Refined grains** | Rice; Traditional Chinese rice-pudding; rice porridge; noodle; macaronis; steamed buns; deep-fried dough stick; biscuits; |
| Potatoes | Potatoes; pueraria thomsonii; taro; Chinese yam; |
| **Sugar sweetened beverages** | Cola; other carbonated beverages with sugar; |
| ***Animal Food Groups*** | |
| **Dairy** | Whole milk; skim milk; whole milk powder; skim milk powder; yogurt; milky tea; milk shake; cheese; ice cream; |
| **Egg** | Eggs |
| **Fish or Seafood** | Fresh water fish; saltwater fish; canned fish; salted fish; mollusks; shrimp; crab; |
| **Meat** | Streaky pork; lean pork; pettitoes; pigskin; beef; mutton; animal organs (stomach, heart, liver, kidney and others); preserved meat; sausage; ham; other processed meat; poultry meat with skin; poultry meat without skin; chicken feet; |

Supplemental Table 2: Associations of plant-based diet index scores with body composition after adjusted for potential covariates in girls and boys.

| Body composition | | Per | | | | | | | | | | |
| --- | --- | --- | --- | --- | --- | --- | --- | --- | --- | --- | --- | --- |
|  |  | PDI | | |  | hPDI | | |  | uPDI | | |
|  |  | *β* | *se* | *p* |  | *β* | *se* | *p* |  | *β* | *se* | *p* |
| **Girls** |  |  |  |  |  |  |  |  |  |  |  |  |
| Whole body | FM, kg | -0.054 | 0.122 | 0.657 |  | -0.125 | 0.126 | 0.319 |  | 0.043 | 0.123 | 0.730 |
|  | LM, kg | 0.115 | 0.123 | 0.351 |  | 0.134 | 0.127 | 0.294 |  | -0.121 | 0.124 | 0.330 |
|  | FMP, % | -0.419 | 0.461 | 0.365 |  | -0.620 | 0.476 | 0.194 |  | 0.301 | 0.468 | 0.521 |
| Trunk | FM, kg | -0.003 | 0.061 | 0.965 |  | -0.029 | 0.063 | 0.646 |  | 0.015 | 0.061 | 0.805 |
|  | LM, kg | 0.050 | 0.065 | 0.445 |  | 0.084 | 0.067 | 0.211 |  | -0.072 | 0.066 | 0.274 |
|  | FMP, % | -0.404 | 0.506 | 0.426 |  | -0.667 | 0.522 | 0.203 |  | 0.427 | 0.513 | 0.406 |
| Limbs | FM, kg | -0.052 | 0.073 | 0.472 |  | -0.091 | 0.075 | 0.224 |  | 0.027 | 0.074 | 0.711 |
|  | LM, kg | 0.066 | 0.070 | 0.349 |  | 0.068 | 0.073 | 0.352 |  | -0.043 | 0.071 | 0.551 |
|  | FMP, % | -0.566 | 0.619 | 0.361 |  | -0.726 | 0.639 | 0.257 |  | 0.246 | 0.628 | 0.695 |
| Android area | FM, kg | 0.006 | 0.010 | 0.538 |  | 0.001 | 0.010 | 0.953 |  | -0.001 | 0.010 | 0.932 |
|  | LM, kg | 0.020 | 0.014 | 0.151 |  | 0.039 | 0.014 | **0.008** |  | -0.018 | 0.014 | 0.213 |
|  | FMP, % | -0.386 | 0.494 | 0.435 |  | -1.072 | 0.505 | **0.035** |  | 0.416 | 0.500 | 0.407 |
| Gynoid area | FM, kg | -0.006 | 0.023 | 0.785 |  | -0.004 | 0.024 | 0.871 |  | 0.006 | 0.023 | 0.802 |
|  | LM, kg | 0.033 | 0.027 | 0.222 |  | 0.062 | 0.028 | **0.027** |  | -0.054 | 0.027 | **0.047** |
|  | FMP, % | -0.614 | 0.544 | 0.261 |  | -0.646 | 0.563 | 0.253 |  | 0.538 | 0.552 | 0.331 |
| **Boys** |  |  |  |  |  |  |  |  |  |  |  |  |
| Whole body | FM, kg | -0.103 | 0.121 | 0.393 |  | -0.217 | 0.149 | 0.145 |  | 0.082 | 0.121 | 0.496 |
|  | LM, kg | 0.048 | 0.116 | 0.681 |  | 0.218 | 0.143 | 0.129 |  | -0.165 | 0.116 | 0.155 |
|  | FMP, % | -0.359 | 0.410 | 0.383 |  | -0.980 | 0.503 | 0.053 |  | 0.522 | 0.409 | 0.203 |
| Trunk | FM, kg | 0.009 | 0.060 | 0.879 |  | -0.067 | 0.074 | 0.368 |  | 0.043 | 0.060 | 0.472 |
|  | LM, kg | 0.009 | 0.057 | 0.875 |  | 0.142 | 0.070 | **0.045** |  | -0.123 | 0.057 | **0.032** |
|  | FMP, % | -0.035 | 0.420 | 0.933 |  | -0.861 | 0.515 | 0.096 |  | 0.646 | 0.417 | 0.123 |
| Limbs | FM, kg | -0.111 | 0.073 | 0.130 |  | -0.141 | 0.090 | 0.119 |  | 0.032 | 0.073 | 0.663 |
|  | LM, kg | 0.043 | 0.068 | 0.522 |  | 0.102 | 0.084 | 0.223 |  | -0.057 | 0.068 | 0.399 |
|  | FMP, % | -0.758 | 0.582 | 0.195 |  | -1.386 | 0.716 | 0.054 |  | 0.567 | 0.582 | 0.331 |
| Android area | FM, kg | -0.006 | 0.012 | 0.610 |  | -0.013 | 0.014 | 0.359 |  | 0.011 | 0.012 | 0.345 |
|  | LM, kg | 0.000 | 0.013 | 0.999 |  | 0.040 | 0.016 | **0.011** |  | -0.025 | 0.013 | **0.050** |
|  | FMP, % | -0.430 | 0.480 | 0.372 |  | -1.51 | 0.586 | **0.010** |  | 1.076 | 0.475 | **0.025** |
| Gynoid area | FM, kg | -0.025 | 0.024 | 0.311 |  | -0.042 | 0.030 | 0.155 |  | 0.028 | 0.024 | 0.255 |
|  | LM, kg | 0.005 | 0.026 | 0.839 |  | 0.040 | 0.033 | 0.225 |  | -0.022 | 0.026 | 0.397 |
|  | FMP, % | -0.569 | 0.533 | 0.287 |  | -1.300 | 0.655 | **0.048** |  | 0.786 | 0.531 | 0.141 |

FM: fat mass; LM: lean mass; FMP: fat mass percentage;

Linear regression analysis, adjusted for covariates including: age, sex, height, weight, delivery way, household income, parental education, physical activity, use of calcium and multi-vitamin supplements, dietary intake of energy.

Supplemental Table 3: Associations of plant-based diet index scores with abdominal obesity.

| Abdominal obesity | Plant-based diet index | | | | | | | | | | | | |
| --- | --- | --- | --- | --- | --- | --- | --- | --- | --- | --- | --- | --- | --- |
|  | *T1* |  | *T2* | | |  | *T3* | | |  | *Per 10 increment* | | |
|  | *Reference* |  | *OR* | *95%CI* | *p* |  | *OR* | *95%CI* | *p* |  | *OR* | *95%CI* | *p* |
| PDI |  |  |  |  |  |  |  |  |  |  |  |  |  |
| Girls | 1.00 |  | 0.48 | (0.09, 2.43) | 0.371 |  | 0.59 | (0.12, 2.96) | 0.525 |  | 0.77 | (0.23, 2.51) | 0.658 |
| Boys | 1.00 |  | 1.61 | (0.49, 5.33) | 0.436 |  | 2.10 | (0.64, 6.90) | 0.222 |  | 1.74 | (0.77, 3.92) | 0.184 |
| hPDI |  |  |  |  |  |  |  |  |  |  |  |  |  |
| Girls | 1.00 |  | 0.47 | (0.11, 1.96) | 0.299 |  | 0.02 | (0.001, 0.48) | **0.016** |  | 0.31 | (0.09, 1.13) | 0.076 |
| Boys | 1.00 |  | 1.26 | (0.38, 4.19) | 0.711 |  | 2.11 | (0.66, 6.77) | 0.208 |  | 1.21 | (0.44, 3.35) | 0.713 |
| uPDI |  |  |  |  |  |  |  |  |  |  |  |  |  |
| Girls | 1.00 |  | 1.48 | (0.28, 7.76) | 0.642 |  | 2.38 | (0.46, 12.2) | 0.299 |  | 1.77 | (0.58, 5.34) | 0.315 |
| Boys | 1.00 |  | 2.79 | (0.70, 11.2) | 0.148 |  | 3.17 | (0.86, 11.7) | 0.084 |  | 1.58 | (0.68, 3.67) | 0.292 |

Logistic regression analysis, adjusted for covariates including: age, sex, height, weight, delivery way, household income, parental education, physical activity, use of calcium and multi-vitamin supplements, dietary intake of energy.
